# Identification of resistance sources and genomic regions regulating spot blotch resistance in Asian bread wheat (*Triticum aestivum* L.) via genome‐wide association study

**DOI:** 10.1002/tpg2.70228

**Published:** 2026-03-27

**Authors:** Nikita Aggarwal, Xinyao He, Mukesh Rathore, Farkhandah Jan, Vikas Gupta, Reyazul Rouf Mir, Pawan K. Singh

**Affiliations:** ^1^ Division of Genetics and Plant Breeding, Faculty of Agriculture SKUAST‐Kashmir Kashmir Jammu and Kashmir India; ^2^ International Maize and Wheat Improvement Center Texcoco Mexico; ^3^ ICAR‐Indian Institute of Wheat and Barley Research Karnal Haryana India; ^4^ Centre for Crop and Food Innovation, WA State Agricultural Biotechnology Centre Murdoch University Murdoch Western Australia Australia

## Abstract

Spot blotch (SB), caused by *Bipolaris sorokiniana*, is a major yield‐limiting disease of wheat (*Triticum aestivum* L.) in the warm, humid agroclimatic zones of South Asia. The development of resistant cultivars through molecular approaches offers a sustainable strategy for managing this disease. This study aimed to identify resistant genotypes and associated single‐nucleotide polymorphism markers in a panel of 187 spring bread wheat lines via field‐based phenotyping and genome‐wide association study (GWAS). The panel was genotyped via the DArTSeq genotyping‐by‐sequencing platform and evaluated for SB resistance under artificially inoculated field conditions across two crop seasons (2019–2020 and 2020–2021) at Agua Fria, Mexico. Significant phenotypic variation was observed, with genotypes BGD54, IND56, and BGD55 showing high levels of resistance, indicating their potential as resistance donors. GWAS identified multiple marker‒trait associations (MTAs) linked to SB resistance, with seven stable MTAs consistently detected across years and models, located on chromosomes 3D, 5A, 3B, and 1B, and explaining 3.86%–18.17% phenotypic variance. Most of these MTAs colocalized with previously reported genomic regions for SB resistance. In silico analysis revealed candidate genes within these regions encoding potassium transporters, zinc finger proteins, glutathione S‐transferases, FBD domain proteins, leucine‐rich repeats, protein kinases, dirigent proteins, and cytochrome P450 enzymes. The identified stable MTAs and associated candidate genes offer valuable resources for marker‐assisted selection and functional validation in wheat breeding programs targeting SB resistance.

## Core Ideas

- Evaluated 187 bread wheat lines for spot blotch resistance across two field seasons.
- BGD54, IND56, and BGD55 identified as promising donors with high resistance.
- GWAS identified seven stable marker‒trait associations on chromosomes 3D, 5A, 3B, and 1B.
- Stable single‐nucleotide polymorphisms explained 3.86%–18.17% of phenotypic variation in disease resistance.
- Several candidate genes linked to defense: leucine‐rich repeats, GSTs, zinc fingers, P450s, and kinases.

## Plain Language Summary

Spot blotch, caused by the fungus *Bipolaris sorokiniana*, is a serious wheat disease that reduces yields, especially in warm and humid regions of South Asia. In this study, 187 wheat varieties from India and Bangladesh were tested for their resistance to the disease under field conditions in Mexico. We identified several wheat lines, such as BGD54, IND56, and BGD55, that showed strong resistance and could be used as parents in breeding programs. Using advanced genetic analysis (genome‐wide association study), we found seven consistent single‐nucleotide polymorphism markers linked to disease resistance, located on four chromosomes. These markers explained up to 18.17% of the variation in resistance and were linked to genes involved in plant defense. These findings provide valuable tools for developing new wheat varieties with durable resistance to spot blotch, helping farmers reduce yield losses and improve food security in regions where the disease is a major problem.

**Abbreviations**
AUDPC: area under the disease progression curve
CIMMYT: International Maize and Wheat Improvement Centre
CMLM: compressed mixed linear model
DH: days to heading
FarmCPU: fixed and random model circulating probability unification
GLM: general linear model
GWAS: genome‐wide association study
LRR: leucine‐rich repeat
MAS: marker‐assisted selection
MLM: mixed linear model
MLMM: multiple loci mixed linear model
MTA: marker–trait association
PH: plant height
PVE: phenotypic variance explained
Q–Q: quantile–quantile
QTL: quantitative trait locus
SB: spot blotch
SNP: single‐nucleotide polymorphism

## 1 INTRODUCTION

Bread wheat (*Triticum aestivum* L.) is one of the world's most important cereal crop species, serving as a major source of calories for the growing global population (P. K. Gupta et al., 2020). The Green Revolution of the 1960s and 1980s significantly increased wheat production, particularly in South Asia (Yadav et al., 2019). However, current production trends are insufficient to meet the demands of the projected global population of nine billion by 2050 (Hemathilake & Gunathilake, 2022). Therefore, increasing wheat yield is crucial for achieving the goal of zero hunger by 2050. To meet this growing demand without expanding cultivated land, which is limited, wheat grain production must be increased. Although production is increasing, yields are still vulnerable to a variety of biotic and abiotic factors. Among biotic stresses, spot blotch (SB) (Helminthosporium leaf blight), caused by the hemibiotrophic fungus *Bipolaris sorokiniana* (Sacc.) Shoemaker [teleomorph: *Cochliobolus sativus* (Ito & Kuribayashi) Drechsler ex Dastur] poses a significant constraint on bread wheat production, particularly in warm and humid regions. This disease threatens the livelihoods of many smallholder farmers (H. J. Dubin & Ginkel, 1991). SB induces foliar necrosis, reducing the photosynthetic area and causing premature senescence (Sharma et al., 1997). Globally, SB affects >25 million ha, representing approximately 12% of the total wheat‐growing area (Duveiller et al., 2005). Yield losses due to SB can range from 0% to 100%, depending on the region and environmental conditions (Juliana, He, Poland, Roy, et al., 2022). With rising global temperatures predicted by climate models, SB is likely to become an even more significant threat to wheat production (P. K. Gupta et al., 2018). More recently, the pathogen has also been documented in temperate environments, such as the Kashmir Valley of the Western Himalayas, indicating a geographical range expansion possibly associated with climate change (Aggarwal et al., 2025). Most high‐yielding wheat varieties in South Asia lack sufficient resistance to SB (Joshi et al., 2007), and the pathogen is highly variable, increasing in aggressiveness over time, which can lead to the breakdown of resistance genes (Aggarwal et al., 2010; Pandey et al., 2008). Consequently, there is a strong need to explore new resistance sources to effectively combat SB disease (Chowdhury et al., 2013; Kaur et al., 2021). Among multifaceted disease control strategies, identifying and deploying resistance genes or loci through breeding programs is a promising and cost‐effective approach for achieving durable and environmentally sustainable control of SB (Maulenbay & Rsaliyev, 2024; Z. Zhu et al., 2014). Developmental traits, such as days to heading (DH) and plant height (PH), are associated with SB resistance but often result in “linkage drag,” which breeders aim to minimize (Duveiller & Sharma, 2009). Understanding the relationships between SB resistance and developmental traits, including DH and PH, is vital for identifying the most relevant resistance loci for specific breeding programs.

Breeding wheat with a combination of climate resilience, disease resistance, and favorable agronomic traits can significantly improve productivity to meet future food demands (Mondal et al., 2016). However, progress in the development of SB‐resistant cultivars has been slow due to the quantitative nature of resistance (Chand et al., 2021; Juliana, He, Poland, Roy, et al., 2022) and the limited number of resistance genes. Currently, four SB resistance genes, *Sb1–Sb4*, have been identified (Kumar et al., 2015; Lillemo et al., 2013; Lu et al., 2016; P. Zhang et al., 2020). The use of molecular markers identified through biparental quantitative trait locus (QTL) mapping and genome‐wide association studies (GWASs) can accelerate the development of resistant cultivars (P. K. Singh et al., 2018). GWAS, which leverages historical recombination, typically provides higher resolution than biparental mapping does and has been increasingly used for gene identification, cloning, functional characterization, and validation. Several complex traits in wheat have been successfully dissected via GWAS, including resistance to Septoria tritici blotch (Miedaner et al., 2024; Patial et al., 2024), Fusarium head blight (Hu et al., 2020; Larkin et al., 2020; Morales et al., 2024), multiple leaf spot diseases (Gurung et al., 2014), tan spot in winter wheat (Kollers et al., 2013; Lhamo et al., 2024), stripe rust (Fatima et al., 2020; Gao et al., 2024; Qiao et al., 2024), Karnal bunt (V. Gupta et al., 2019; S. Singh et al., 2020), leaf rust (Leonova et al., 2020; Vikas et al., 2022), and powdery mildew (Kang et al., 2020; G. Li et al., 2019).

Numerous marker‒trait associations (MTAs) for SB resistance have been identified across various chromosomes (Ayana et al., 2018; Bainsla et al., 2020; Jamil et al., 2018; Juliana, He, Poland, Roy, et al., 2022; Kamble et al., 2024; Lozano‐Ramirez et al., 2022; Tomar et al., 2020). Despite these advances, the currently identified genes, QTLs, and MTAs do not represent the full spectrum of loci that may contribute to SB resistance. There is significant potential for discovering novel MTAs via diverse germplasms. Since the 1990s, the International Maize and Wheat Improvement Centre (CIMMYT) has intensified efforts to screen and breed for SB resistance (Dubin & Rajaram, 1996). Elite breeding lines combining strong SB resistance and high yield potential have been compiled in the Helminthosporium Leaf Blight Screening Nursery.

In this study, we conducted a GWAS using five models to identify resistant donors, uncover novel MTAs, and predict potential candidate genes in a panel of bread wheat genotypes collected from India and Bangladesh. As anticipated, several novel MTAs involved in SB resistance were identified. We expect these findings to provide a valuable foundation for breeders to increase durable resistance to SB in future wheat cultivars.

**Core Ideas**

- Evaluated 187 bread wheat lines for spot blotch resistance across two field seasons.
- BGD54, IND56, and BGD55 identified as promising donors with high resistance.
- GWAS identified seven stable marker‒trait associations on chromosomes 3D, 5A, 3B, and 1B.
- Stable single‐nucleotide polymorphisms explained 3.86%–18.17% of phenotypic variation in disease resistance.
- Several candidate genes linked to defense: leucine‐rich repeats, GSTs, zinc fingers, P450s, and kinases.

## 2 MATERIALS AND METHODS

### 2.1 Association mapping panel and field layout

The SB Association Mapping Panel consisted of 187 bread wheat (*Triticum aestivum* L.) accessions (Table S1) sourced from wheat breeding programs in India (94 genotypes) and Bangladesh (93 genotypes). Field screening experiments were conducted at CIMMYT's Agua Fria Experimental Station in the State of Puebla, Mexico (altitude: 100 m, latitude: 20.5° N, longitude: 97.6° W), a well‐known hotspot for SB disease. The location experiences warm and humid conditions during the wheat‐growing season from November to March, with an average annual rainfall of 1200 mm, making it highly conducive to SB epidemics.

The panel was evaluated during the 2019–2020 and 2020–2021 growing seasons, designated SB20 and SB21, respectively. The plant materials were sown in 1‐m row plots in a randomized complete block design with two replications. The wheat genotypes Chirya 3 and Ciano T79 were used as resistant and susceptible controls, respectively.

### 2.2 Trait phenotyping for SB analysis

The inoculum consisted of a composite mixture of five aggressive *B. sorokiniana* isolates maintained at the CIMMYT Wheat Pathology Laboratory, El Batán. These virulent isolates, previously stored at −20°C, were reactivated and cultured on V8 media for 5–7 days at 22°C–25°C to promote mycelial growth. Subsequently, the cultures were propagated on autoclaved sorghum seeds. The inoculated sorghum grains were placed in flasks and incubated at room temperature for 6 weeks with regular shaking to ensure uniform fungal colonization. The isolates had been previously characterized for high virulence and were combined in equal proportions to ensure broad and uniform disease pressure. The same mixed inoculum was used consistently across both cropping seasons (2019–2020 and 2020–2021) to maintain experimental uniformity. Although the experimental site represents a natural hotspot for SB, artificial inoculation was employed to ensure uniform and sufficient disease pressure across genotypes and seasons, as natural infection alone can be inconsistent and unreliable for genetic association studies. At the Zadoks' Growth Stage 29, the infected sorghum grains were spread in the center of two rows (Zadoks et al., 1974). Four to 5 weeks after inoculation, disease severity was visually assessed in each plot via the two‐digit scale (00–99) described by Saari & Prescott (1975). The first digit (D1) represents the vertical progression of the disease in the canopy, whereas the second digit (D2) indicates the severity of the disease, that is, the percentage of leaf area affected. Both D1 and D2 were scored on a 1–9 scale. Disease evaluations were performed four times at 7‐day intervals.

For each evaluation, the disease severity percentage was calculated via the formula % severity = (D1/9) × (D2/9) × 100, which was then used to calculate the area under the disease progression curve (AUDPC). The mean AUDPC values from two replicates in each year, as well as the average values across 2 years, were used for GWASs.

### 2.3 Agronomic data scoring

PH and DH were recorded in all field experiments. PH was measured at physiological maturity, from the ground to the average spike tips, excluding awns. DH was determined as the number of days from sowing to the point when approximately 75% of the spikes had emerged. To investigate the associations between DH, PH, and SB resistance, these traits were scored across 2 years.

### 2.4 Genotyping, population structure, and linkage disequilibrium

This panel was genotyped in our previous study on Septoria tritici blotch (Patial et al., 2024) via the DArTSeq genotyping‐by‐sequencing platform (H. Li et al., 2015). The average executed sequencing depth per locus was 7.3x, calculated as the mean sum of sequence counts for both reference and single‐nucleotide polymorphism (SNP) alleles across all targeted loci. Markers were filtered on the basis of a minor allele frequency (MAF) >5%, missing data <30%, and heterozygosity <10% for further analysis. The population structure and linkage disequilibrium (LD) were analyzed as described in Patial et al. (2024).

### 2.5 Identification of MTAs

GWAS were performed via the GAPIT package version 3.0 in R software (Wang & Zhang, 2021) to detect significant MTAs. Five association models were implemented for this study: the general linear model (GLM) (Bradbury et al., 2007), mixed linear model (MLM) (Z. Zhang et al., 2010), multiple loci mixed linear model (MLMM) (Segura et al., 2012), compressed mixed linear model (CMLM) (Z. Zhang et al., 2010), and fixed and random model circulating probability unification (FarmCPU) (Liu et al., 2016). These models differ in their structures, with all except GLM incorporating mixed‐effect components, including both fixed and random effects. The MLMM and FarmCPU models are designed for multi‐locus analysis, with the FarmCPU model being particularly effective at controlling both false positives and false negatives (Liu et al., 2016; Kaler & Purcell, 2019).

The analysis of SB resistance was conducted for individual years, as were the best linear unbiased estimator means across years. Additionally, a GWAS was performed on PH and DH to investigate potential associations with SB resistance. All MTAs with a stringent logarithm of odds ≥ 3 (−log10 of the *p* value) were considered significant for SB resistance. Manhattan and quantile–quantile (*Q*–*Q*) plots were generated via the CMplot‐R tool in RStudio.

### 2.6 Identification of putative candidate genes

To identify potential candidate genes associated with significant MTAs, SNP sequences were subjected to BLAST searches against the wheat reference genome sequence IWGSC (RefSeq v1.0) available in Plant Ensembl (https://plants.ensembl.org/Triticum_aestivum/Info/Index). This step aimed to retrieve the corresponding genes and their functional descriptions. For precise candidate gene identification, marker physical locations and chromosome names were directly entered into Ensembl Plants.

Markers with SNPs residing within gene sequences were classified as “direct gene hits.” For markers lacking SNPs within genes, potential candidate genes were selected within ± 1 Mb upstream and downstream (2 Mb window) of the marker location. In instances where annotations for genes were unavailable in the *Triticum aestivum* genome, the comparative genomics tool in Ensembl was employed to identify orthologous genes in related species with known predicted functions. Additionally, previously published reports were consulted to evaluate the role of these putative candidate genes in regulating desired traits.

### 2.7 Statistical analysis

R software was used for all data analysis. Analysis of variance (ANOVA) was performed to assess variation in disease severity, followed by a least significant difference test to compare significant mean values. Phenotypic and genotypic coefficients of variation, along with broad‐sense heritability, were estimated via the formula described by Singh and Chaudhary (1981).

$$H^{2}=\sigma_{g}^{2}/\left(\sigma_{g}^{2}+\sigma_{g\times y}^{2}/y+\sigma_{e}^{2}/\mathrm{ry}\right)$$

where $\sigma_{g}^{2}$ represents genetic variance, $\;\sigma_{g\;\times \;y}^{2}$ represents variance due to genotype‐by‐year interaction, $\sigma_{e}^{2}$ represents experimental error, and *y* and *r* represent the number of years and replications, respectively. Pearson's correlation coefficients between SB (AUDPC) and agronomic variables were determined via SAS software version 9.2.

## 3 RESULTS

### 3.1 Phenotypic evaluation and heritability

**FIGURE 1:**
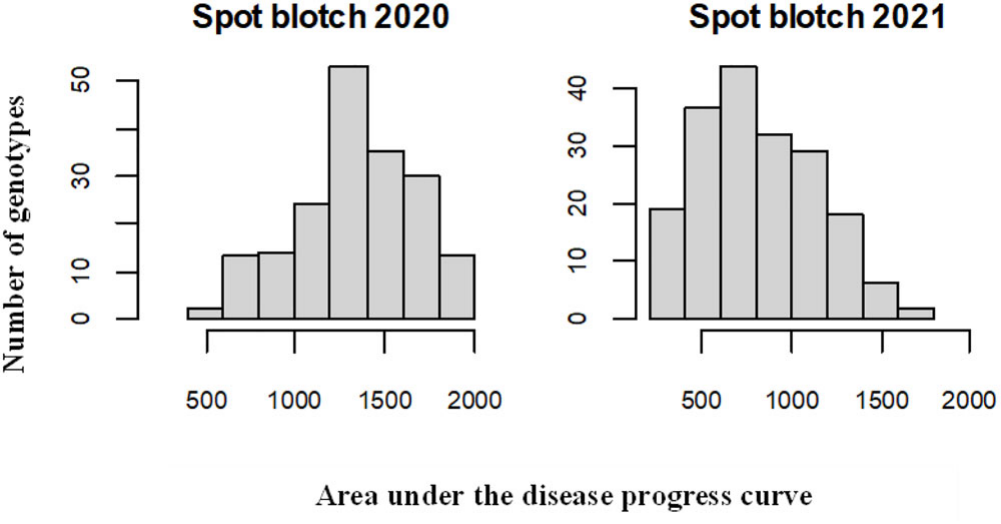
Histograms of spot blotch severity (area under the disease progression curve [AUDPC]) in individual experiments.

The 187 wheat genotypes evaluated for SB severity presented a wide range of AUDPC scores. In 2020, the AUDPC scores ranged from 546.29 to 1995.37, whereas in 2021, they ranged from 263.58 to 1603.08, indicating substantial phenotypic variation with a continuous distribution of lines in both years (Figure 1; Table S1).

Among the tested entries, BGD54, IND56, BGD55, BGD51, and IND84 presented high levels of resistance to SB, with mean AUDPC scores of 409.3, 415.1, 455.9, 538.9, and 541.7, respectively (Table 1). In contrast, IND26, IND60, IND61, IND79, and IND94 were the most susceptible, with mean AUDPC scores of 1784.1, 1743.1, 1725.8, 1685.8, and 1683.6, respectively (Table S1). The AUDPC for the resistant check Chirya 3 was recorded as 565.7, whereas for the susceptible check Ciano T79, it was 1613.4 (Table 1). Interestingly, five out of the six best Bangladesh lines have Chirya 7 and/or Chirya 1, two sister lines of Chirya 3, in their pedigrees (Table 1).

**TABLE 1:** Top resistant lines to spot blotch identified in the 2020 and 2021 experiments.

Code	Name or pedigree	DH	2020	2021	Mean
BGD54	CHIR7/ANB//CHIR1	65.5	546.3	272.2	409.3
IND56	KRL 213	69.7	558.0	272.2	415.1
BGD55	CHIR7/ANB//CHIR1	66.7	639.5	272.2	455.9
BGD51	CHIR7/ANB//CHIR1	67	746.9	332.7	539.8
IND84	UP 2942	68.7	759.3	324.1	541.7
BGD53	CHIR7/ANB//CHIR1	66.2	765.7	347.8	556.8
IND8	DBW 168	64.5	938.0	324.1	631.0
BGD57	BIJOY/SHA3/4/SERI//SHA4/LIRA/3/CHIR1	67	893.8	375.9	634.9
IND4	DBW 88	65.7	824.4	484.0	654.2
IND88	UP 2993	63	951.2	406.2	678.7
IND20	DBW 248	63.5	781.5	576.9	679.2
IND22	DBW 252	64.7	858.0	518.5	688.3
IND35	HD 3184	68.7	978.1	445.1	711.6
IND7	DBW 150	68.7	926.5	512.0	719.3
BGD88	CAL/NH//H567.71/3/SERI/4/CAL^a^	67.5	1012.0	464.5	738.3
R Check	Chirya 3	75.5	664.8	466.7	565.7
S Check	Ciano T79	56	1898.1	1328.7	1613.4

*Note*: Lines with days to heading (DH) >70 days are not shown due to their disease escape mechanism.

^a^ Pedigree information is incomplete; refer to Table S1 for the full pedigree information.

A combined analysis of variance over 2 years revealed that the effects of genotype, year, and their interaction were highly significant for SB severity (Table 2). “Year” was the greatest source of variation, followed by “genotype.” The broad‐sense heritability for SB severity was estimated at a high value of 0.90. The SB severity data exhibited a high correlation between the 2 years, with a Pearson's correlation coefficient of 0.84. DH was consistently significantly associated with SB severity, ranging from −0.49 to −0.69, whereas PH was not significantly associated with SB severity.

**TABLE 2:** Analysis of variance for spot blotch in the genome‐wide association study (GWAS) panel.

Source	DF	Mean square	*F* value	*p* value
Genotype	186	378,818.4	51.83	<0.0001
Year	1	52,927,909.0	7241.57	<0.0001
Rep (year)	2	217,423.6	29.75	<0.0001
Genotype × year	183	36,463.4	4.99	<0.0001
Error	369	7308.9		

### 3.2 SNP density and principal component analysis

**FIGURE 2:**
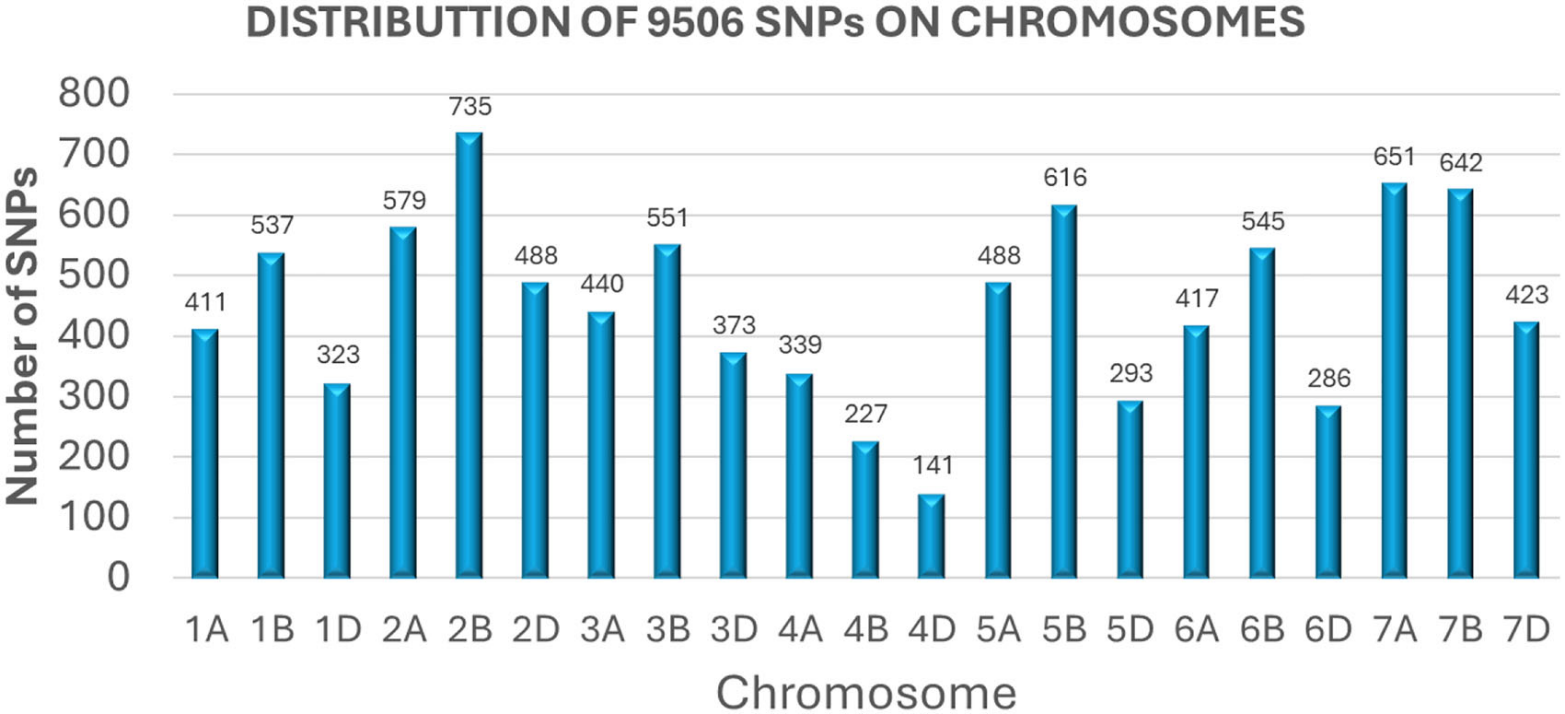
Distribution of single‐nucleotide polymorphisms (SNPs) over 21 wheat chromosomes.

Genotyping yielded a total of 50,000 SNPs, which were subsequently filtered to remove low‐quality markers, resulting in 9506 polymorphic SNPs. These SNPs were distributed genome‐wide across all 21 bread wheat chromosomes, with SNP counts per chromosome ranging from 141 SNPs on chromosome 4D to 735 SNPs on chromosome 2B, averaging 452.7 SNPs per chromosome (Figure 2).

**FIGURE 3:**
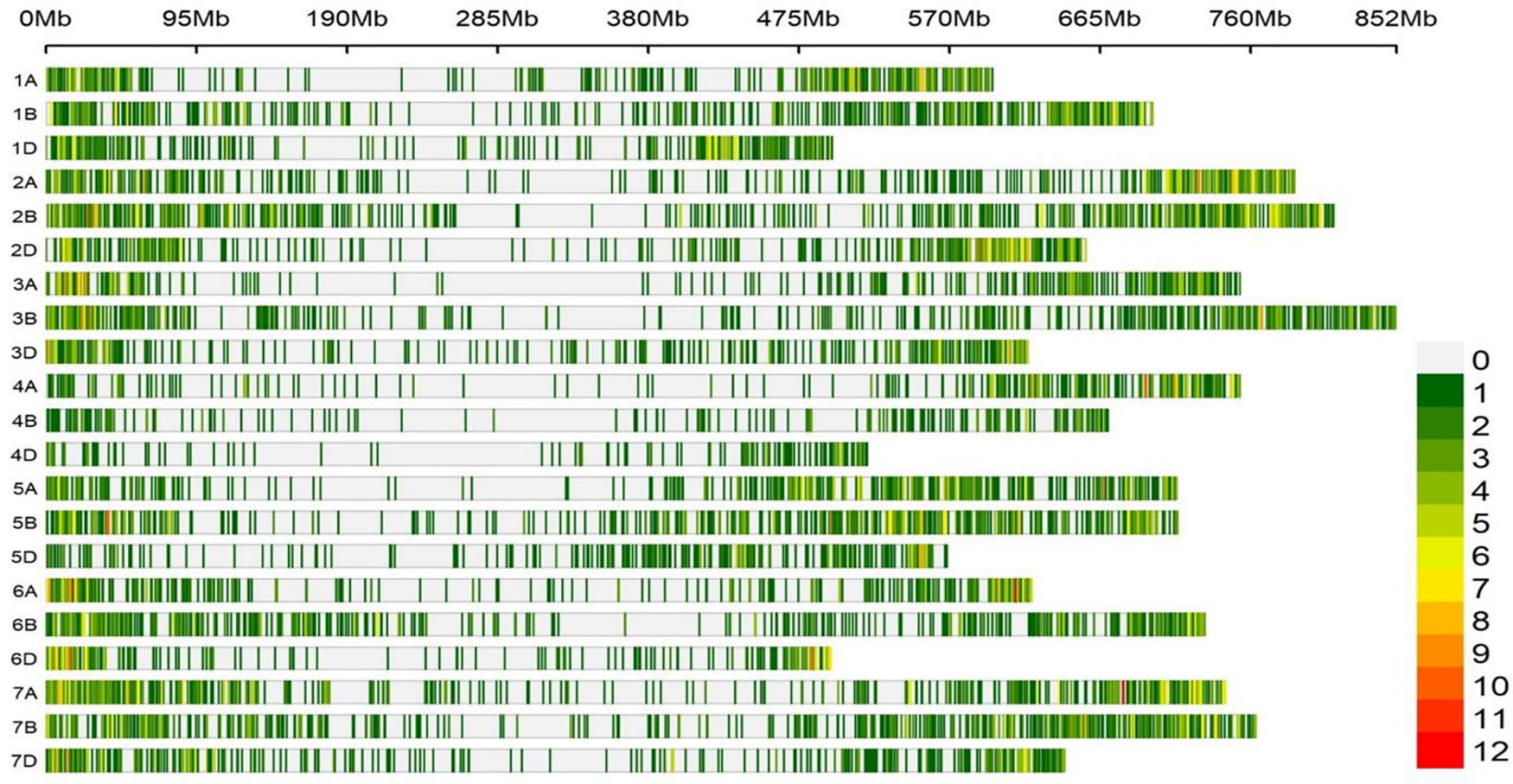
Single‐nucleotide polymorphism (SNP) density plot, chromosome‐wise, representing number of SNPs within 1 Mb window size. The horizontal axis shows the chromosome length (Mb); the different color depicts SNP density.

When the SNP density (SNPs per megabase) on each chromosome was analyzed, the SNP density ranged from 1.1 SNPs/Mb on chromosome 3D to 3.67 SNPs/Mb on chromosome 4D, with an overall average of 1.64 SNPs/Mb (Figure 3). Detailed information regarding population structure and LD can be found in a previous publication using the same panel (Patial et al., 2024).

### 3.3 MTAs for SB analysis

GWAS for SB resistance was conducted on the basis of the AUDPC scores collected over two consecutive years. A significance threshold of *p* < 0.001 was used to identify MTAs. Various GWAS models led to the identification of 321, 19, 20, 17, and 16 MTAs in 2020 and 331, 29, 16, 12, and 11 MTAs in 2021 through GLM, FarmCPU, MLMM, CMLM, and MLM, respectively. When the pooled data across both environments were analyzed, the maximum number of MTAs was identified via GLM (21), followed by MLMM (14), CMLM and MLM (11 each), and FarmCPU (5) (Tables S2–S6).

**FIGURE 4:**
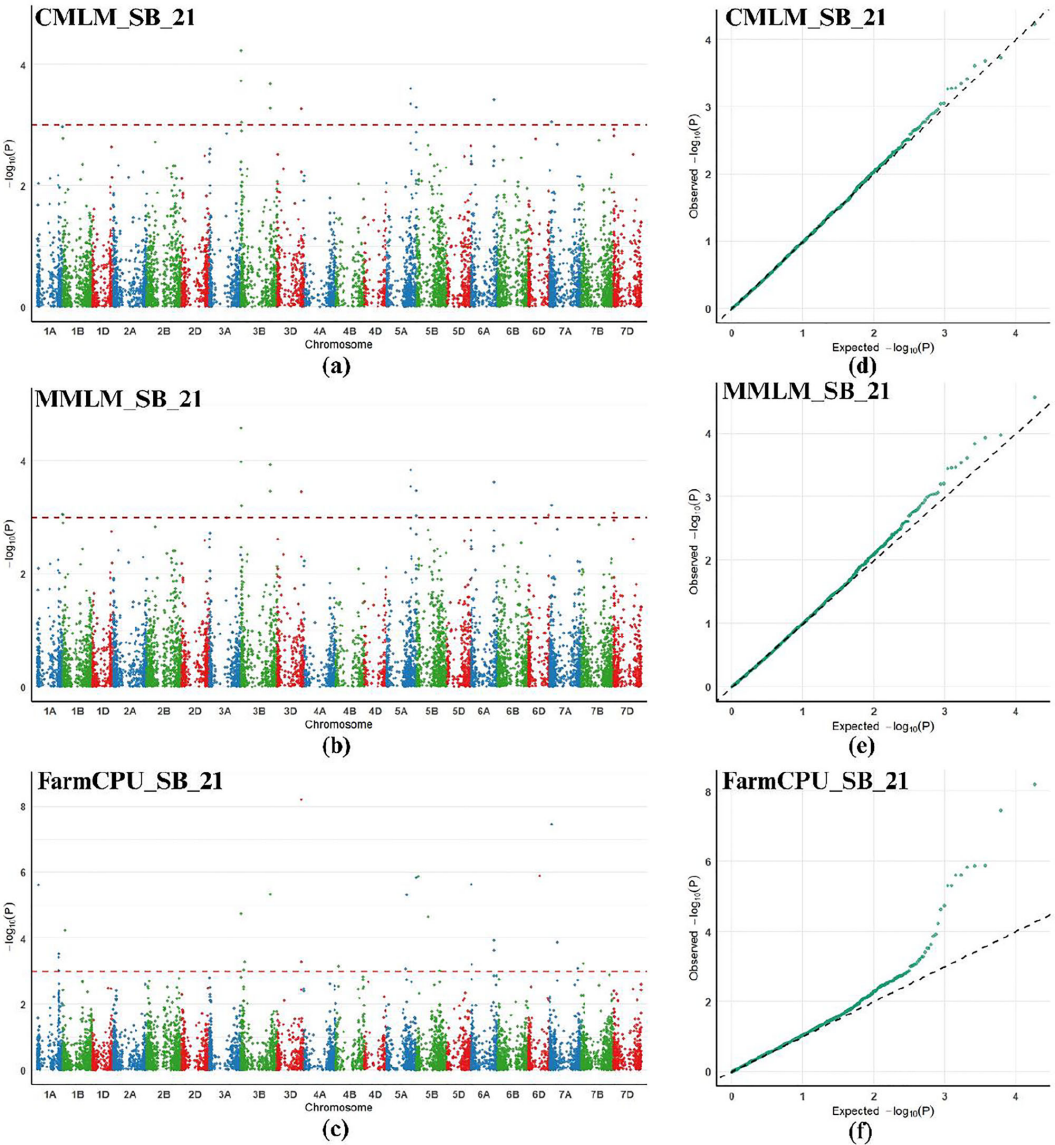
Manhattan and *Q*–*Q* plots for spot blotch resistance generated using the compressed mixed linear model (CMLM), multiple loci mixed linear model (MLMM), and fixed and random model circulating probability unification (FarmCPU) model in environment SB21.

To ensure reliability, we mined MTAs that were consistent across both environments and detected by at least two models, which we considered stable. This process resulted in the identification of seven stable MTAs, including SNPs 1,695,432 and 994,016, which are common across all five models, and SNPs 1,091,615 and 5,411,850, which were identified in four models. Additionally, SNPs 1,037,234, 1,003,923, and 1,090,465 were detected in the two models across both environments. These seven MTAs were distributed across four chromosomes: 3D, 5A, 3B, and 1B. The phenotypic variation explained by these stable associations ranged from 3.86% to 18.17%. Two MTAs had positive effects, and five had negative effects on SB resistance (Table 3). Manhattan plots and *Q*–*Q* plots were used to visualize the significant markers (Figure 4; Figures S1–S3).

**TABLE 3:** Information for stable marker‐trait associations for spot blotch identified via genome‐wide association study (GWAS).

S.no.	Traits	Env.	SNP	Chromosome	Position	Model	*p* value range	maf	*R* ^2^ value range
**1**	Spot blotch	SB20	1,695,432	3D	561,275,268	GLM, CMLM, MLM, MLMM, FarmCPU	3.26–8.19	0.090909091	3.94–15.81
		SB21				GLM, CMLM, MLM, MLMM, FarmCPU			
**2**		SB20	994,016	5A	705,576,477	GLM, CMLM, MLM, MLMM, FarmCPU	3.28–8.45	0.112299465	3.96–18.17
		SB21				GLM, CMLM, MLM, MLMM, FarmCPU			
**3**		SB20	1,091,615		569,892,433	GLM, CMLM, MLM, MLMM	3.06–3.83	0.441176471	3.86–6.09
		SB21				GLM, CMLM, MLM, MLMM			
**4**		SB20	5,411,850	3B	684,334,097	GLM, CMLM, MLM, MLMM	3.27–6.62	0.085561497	3.95–15.02
		SB21				GLM, CMLM, MLM, MLMM			
**5**		SB20	1,037,234		681,714,448	GLM, FarmCPU	3.68–9.43	0.106951872	4.55–17.71
		SB21				GLM, CMLM, MLM, MLMM, FarmCPU			
**6**		SB20	1,003,923		1,295,732	GLM, FarmCPU	3.34–6.88	0.323529412	13.73–15.72
		SB21				GLM, FarmCPU			
**7**		SB20	1,090,465	1B	55,315,950	GLM, MLMM	3.01–8.42	0.379679144	15.56–18.11
		SB21				GLM, FarmCPU			

Abbreviations: CMLM, compressed mixed linear model; Env., Environment; FarmCPU, fixed and random model circulating probability unification; GLM, general linear model; MLM, mixed linear model; MLMM, multiple loci mixed linear model; SNP, single‐nucleotide polymorphism.

### 3.4 DH and PH

Similarly, a GWAS was also conducted for DH and PH, and the models identified numerous MTAs on different chromosomes (Tables S7–S16). The most stable MTAs for DH were SNP‐5,411,867, 4,404,915, and 1,106,357, which are all located on chromosome 5A, with phenotypic variation ranging from 4.35% to 14.91%. For PH, the most stable MTAs were SNP‐992,845 on chromosome 5B and SNP‐1,208,202 on chromosome 7D, with phenotypic variation ranging from 5.5% to 12.02% (Table S17).

### 3.5 Candidate genes for MTAs

The significant and stable SNPs associated with SB, DH, and PH were utilized to identify putative candidate genes by alignment with the wheat reference genome IWGSC RefSeq v1.0, as presented in Table S18. SNPs (1,695,432, 994,016, 1,091,615, 5,411,850, 1,037,234, 1,003,923, and 1,090,465) were located in the vicinity of genes encoding proteins with diverse functions, such as F‐box domain‐containing proteins, potassium transporters, zinc finger proteins, glutathione S‐transferase, FBD domain proteins, leucine‐rich repeat (LRR)‐containing proteins, protein kinases, dirigent proteins, UDP‐glucuronosyl/UDP‐glucosyltransferases, thioesterase domain‐containing proteins, glycoside hydrolase family 1 proteins, chitin‐binding proteins (type 1), and cytochrome P450 enzymes. Gene Ontology analysis classified these candidate genes into three major functional categories: molecular function, biological process, and cellular component.

## 4 DISCUSSION

Although phenotype‐based selection in conventional breeding has significantly improved wheat yield over several decades, genotype‐based strategies can further enhance varietal improvement programs. Recent advancements in wheat genome sequencing offer the potential for accelerated variety improvement through molecular breeding, leveraging genetic resources. Numerous QTLs and MTAs have been identified for disease resistance in wheat; however, additional genetic studies using diverse germplasms are necessary, as the saturation point for genetic discovery has not yet been reached (K. Singh et al., 2021). Given the genetic complexity of the wheat genome, there remains a strong possibility of identifying novel QTLs from new genetic materials. In this study, a panel of bread wheat genotypes from India and Bangladesh was evaluated for field resistance to SB. On the basis of these results, several resistant and moderately resistant genotypes were identified, with the panel demonstrating significant variation in SB resistance. These resistant lines, which originate from different regions, represent diverse sources of resistance and can serve as valuable donors for SB resistance breeding. The continuous distribution of disease scores across experiments suggests that resistance is quantitative and governed by the additive effects of multiple QTLs and genes (Ayana et al., 2018; Joshi et al., 2004; Kumar et al., 2007; Neupane et al., 2007; Singh et al., 2018; Singh et al., 2023). ANOVA revealed significant effects of year and genotype‐by‐year interactions, underscoring the importance of evaluating germplasms across multiple years and locations in disease hotspots to identify durable and stable SB‐resistant genotypes (Chattopadhyay et al., 2022; Roy et al., 2021; Singh et al., 2015).

The genetic variance and broad‐sense heritability estimated across environments were high, which is consistent with findings from previous studies (Joshi et al., 2004; Sharma et al., 1997). Resistance to SB was negatively correlated with PH and DH, which are considered confounding factors. Several studies have reported increased disease severity in earlier heading and dwarf genotypes (Sharma et al., 1997; H. J. Dubin et al., 1998; Sharma R.C. et al., 2006; P. K. Saxena et al., 2017; Singh et al., 2015; Singh et al., 2018). This relationship suggests that genotypes with taller and later phonologies might escape SB infection or that their resistance genes may exhibit pleiotropic effects or be tightly linked to SB resistance genes. For traits that are negatively associated, breeding strategies aimed at breaking undesirable linkages are recommended to enable independent improvement of the traits.

Although the size of the association panel used in this study was moderate (187 genotypes), several factors contributed to strong statistical power for detecting meaningful MTAs for SB resistance. The high broad‐sense heritability observed (*H* ^2^ = 0.90) indicates that phenotypic variation was largely under genetic control. Moreover, precise phenotyping under artificial inoculation at a well‐established SB hotspot, combined with evaluation across two growing seasons, reduced environmental noise and improved the reliability and stability of the detected associations. The application of multiple GWAS models further improved detection power while effectively limiting false‐positive associations. Markers with a MAF below 5% were excluded, indicating that the study was primarily designed to detect common genetic variants with moderate effects rather than rare variants. Comparable population sizes have been successfully used to identify stable SB resistance loci in wheat GWAS studies (Jamil et al., 2018; Kamble et al., 2024; Navathe et al., 2022; Tomar et al., 2021).

Using five different GWAS models with a *p* value threshold of 0.001, several significant SNPs were identified. Among them, seven MTAs were consistently detected across both environments and by more than one model for SB resistance. These stable MTAs are promising candidates for further validation in different genetic backgrounds, with potential use in marker‐assisted selection (MAS).

Three of the seven stable MTAs (SNPs 5,411,850, 1,037,234, and 1,003,923) for SB resistance were identified on chromosome 3B and mapped at 684.3 Mb, 681.7 Mb, and 1.2 Mb, respectively, with phenotypic variance explained (PVE) ranging from 3.95% to 17.71%. The marker AX‐94529408, located on chromosome 3BL at 719.8 Mb, was successfully validated through the KASP marker by Kumar et al. (2022) and is located in close proximity to the SNPs identified in this study. Furthermore, Kamble et al. (2024) identified two SNPs on chromosome 3B within the genomic region of 6.5 Mb–6.6 Mb. One of the *Sb* resistance genes, *Sb3*, has been well mapped on 3B and plays a significant role in conferring resistance to SB (Lu et al., 2016). Another study by Juliana, He, Poland, Roy, et al. (2022) identified 16 markers on chromosome 3B that were significantly associated with SB in multiple panels. Bainsla et al. (2020) also identified significant SNPs on chromosome 3B, which were mapped at different locations, confirming the importance of this chromosome in SB resistance.

Chromosome 5A also harbors important loci for SB resistance. Two stable MTAs, SNP 994,016 at 705.5 Mb and SNP 1,091,615 at 569.8 Mb, were identified, with PVE values ranging from 3.86% to 18.17%. Previously, Kamble et al. (2024) identified four consistently significant markers on chromosome 5A, with three located at 414.4 Mb and one MTA (Kukri_c6266_260) located at 607.6 Mb, which is not very far (20.23 Mb) from *Vrn‐A1*, a gene for DH and is often associated with field resistance to SB (He et al., 2021; Singh et al., 2018). Additionally, Bainsla et al. (2020) identified significant SNPs at 569.6 Mb and 586.6 Mb, which are very close to our identified SNP at 569.8 Mb. In the present study, three stable MTAs (SNPs 5,411,867, 4,404,915, and 1,106,357) for DH were identified on chromosome 5A and mapped at 588.4 Mb, 588.1 Mb, and 590.2 Mb, respectively, with PVE values ranging from 4.35% to 14.91%. This chromosome region (588.1–590.2 Mb) contains the *Vrn‐A1* gene (587.4 Mb), implying that the MTAs in this region identified in the current and previous studies for SB resistance might not be caused by the existence of resistance genes but rather by the confounding effects of DH on SB.

Another significant SNP, 1,695,432, was detected on chromosome 3D at 561.2 Mb via all five GWAS models, with PVE ranging from 3.94% to 15.81%. In a previous study by Kumar et al., 2022, another marker, AX‐94794021, was identified on chromosome 3D and mapped at 573.1 Mb, which is close to the SNP identified in this study. The SNP 1,090,465 on chromosome 1B, mapped at 55.3 Mb, with a PVE ranging from 3.01% to 8.42%, represents a potentially novel MTA for SB resistance, as no prior studies have identified MTAs at this position. However, other studies have reported MTAs on chromosome 1B in different genomic regions (Bainsla et al., 2020; Gurung et al., 2014; Juliana, He, Poland, Roy, et al., 2022; Kamble et al., 2024).

Nevertheless, close linkage or coincidence does not necessarily imply that the identified regions represent the same QTL or MTA, especially since this study screened unexplored germplasm for SB resistance. Therefore, the regions discussed above might harbor novel alleles. However, their localization on chromosomes previously reported to contain SB‐associated MTAs suggests that these regions represent important targets for resistance and should be considered critical for future breeding programs aimed at improving SB resistance.

It is important to note that resistance to necrotrophic pathogens such as *B. sorokiniana* is not governed solely by classical resistance genes (P. K. Gupta et al., 2023). In necrotrophic interactions, disease development is often mediated by dominant host susceptibility genes interacting with pathogen‐derived effectors, as exemplified by the well‐characterized ToxA‐Tsn1 interaction, originally characterized in wheat‐tan spot (*Pyrenophora tritici‐repentis*) and wheat‐Septoria nodorum blotch (*Parastagonospora nodorum*) systems. More recently, studies have shown that *B. sorokiniana* produces the necrotrophic effector ToxA and that the ToxA–Tsn1 interaction plays a significant role in SB development on *Tsn1*‐containing wheat genotypes (Manan et al., 2023; McDonald et al., 2018). In the present study, no stable MTAs were detected at or near the *Tsn1* locus on chromosome 5BL. Although a few environment‐specific associations were observed on chromosome 5B, their lack of stability suggests that the major MTAs identified in this study are largely independent of the classical *Tsn1*‐mediated susceptibility pathway. Importantly, a parallel study conducted at the same location and using the same inoculum source identified *Tsn1* as a significant locus influencing SB severity, indicating that the pathogen population used likely harbors an active ToxA effector (Kamble et al., 2024). Therefore, the absence of a comparable *Tsn1* signal in our analysis cannot be attributed to the absence of ToxA activity in the pathogen population. Marker‐based screening of the association panel (see Table S19) indicated that approximately 11% of accessions (∼21 out of 188) carried the *Tsn1* allele. While this relatively low frequency may have reduced statistical power to detect consistent associations at this locus across environments and models, allele frequency alone may not fully account for the absence of a stable *Tsn1* signal. Notably, similar observations have been reported in other necrotrophic wheat pathosystems. For example, Phan et al. (2018) conducted a GWAS using the Vavilov wheat collection infected with *P. nodorum* strain carrying ToxA, Tox1, and Tox3. Despite the presence of *Tsn1* in the wheat panel and ToxA in the pathogen, the ToxA–Tsn1 interaction was not detected, whereas Tox1–Snn1 and Tox3–Snn3 associations were significant. The authors proposed that this pattern reflected effector epistasis, whereby one necrotrophic effector‐host interaction can suppress or mask the phenotypic contribution of another. Similar regulatory interactions have been observed in *P. tritici‐repentis*, where deletion of ToxA increased virulence on some ToxA‐sensitive wheat cultivars and unmasked a previously suppressed chlorosis‐inducing factor (Manning & Ciuffetti, 2015; Moffat et al., 2014). Mechanistic studies summarized by Tan and Oliver (2017) further illustrate that such epistatic interactions are common in the wheat‐*P. nodorum* and wheat‐*P. tritici‐repentis* pathosystems. Given the functional similarities among necrotrophic wheat pathogens, a comparable effector epistasis mechanism may operate in the *B. sorokiniana*‐wheat interaction observed in this study.

Collectively, the stable MTAs identified here likely represent genomic regions contributing to SB resistance through Tsn1‐independent mechanisms, potentially involving additional as yet uncharacterized susceptibility or quantitative resistance loci. Given the likelihood that additional necrotrophic effectors may contribute to *B. sorokiniana* virulence (Kaladhar et al., 2023; McDonald et al., 2018; W. Zhang et al., 2022), these results highlight the inherent complexity of breeding for durable SB resistance. Our findings support integrating the elimination of major susceptibility genes, such as *Tsn1*, with the deployment of GWAS‐identified resistance loci, while accounting for effector epistasis in future studies and breeding programs. Such an integrated strategy is critical for achieving durable and broad‐spectrum resistance against SB in wheat.

Several protein‐coding genes associated with disease resistance were identified through stable MTAs. The identification of candidate genes in GWAS is influenced by the extent of LD surrounding associated markers. In hexaploid wheat, LD often extends over several megabases due to its large genome size and polyploid nature (Pang et al., 2020; Roncallo et al., 2021). In the present study, genome‐wide LD decayed to half of its maximum at approximately 4.9 Mb, with even slower decay observed in the B (5.08 Mb) and D (7.13 Mb) subgenomes (Patial et al., 2024). Based on this LD decay pattern, a ± 1 Mb window around stable MTAs was selected as a conservative physical interval for candidate gene identification. This approach prioritizes genes in close physical proximity to associated markers while limiting the inclusion of large numbers of unrelated loci. Smaller windows may exclude biologically relevant genes located within extended LD blocks, whereas substantially larger intervals could reduce confidence by incorporating excessive non‐causal candidates. Similar window sizes have been employed in several wheat GWAS studies for candidate gene identification (Nouraei et al., 2024; Wang et al., 2021; Xu et al., 2024). A comprehensive literature review highlighted specific candidate genes of interest that are potentially involved in plant defense mechanisms against pathogen infections. For example, F‐box family proteins mediate various biological processes, including leaf senescence (Woo et al., 2001) and responses to both biotic (Kim & Delaney, 2002) and abiotic stresses (Calderón‐Villalobos et al., 2007). The identified SNPs harbor zinc finger domains and RING/U‐box superfamily proteins, which are known to play key roles in resistance to fungal pathogens (Ayana et al., 2018).

We also identified glutathione‐S‐transferases, which are typically upregulated to combat the effects of excessive hydrogen peroxide production during infection (Powell et al., 2017). LRR containing proteins are central components of the plant innate immune system and function as pathogen recognition receptors (Tang et al., 2019). In plants, LRR domains are commonly found in receptor‐like kinases (LRR‐RLKs), receptor‐like proteins (LRR‐RLPs), and intracellular nucleotide‐binding site‐LRR (NBS‐LRR or NLR) proteins. These receptors perceive pathogen‐associated molecular patterns (PAMPs) or effectors to activate PAMPs‐triggered or effector‐triggered immunity. Upon pathogen recognition, LRR‐containing proteins initiate downstream defense responses such as calcium influx, reactive oxygen species accumulation, activation of MAPK cascades, and transcriptional reprogramming of defense‐related genes (Noman et al., 2019). Previous studies have demonstrated the involvement of LRR‐containing proteins in resistance against a wide range of pathogens in wheat (Cao et al., 2024; Jha et al., 2026; Sun et al., 2023).

Additionally, studies have demonstrated that protein kinase‐induced defense signaling downregulates photosynthesis‐related proteins while promoting the accumulation of reactive oxygen species, such as superoxide and hydrogen peroxide, which increase pathogen resistance (Barka et al., 2023). Gene expression analysis has shown that many dirigent and dirigent‐like genes are stress inducible, with upregulation observed during insect (Ralph et al., 2007) and fungal (L. Zhu et al., 2007) attacks. Glycoside hydrolase proteins are involved in a wide range of processes, including starch metabolism, transport, stress defense, and cell wall remodeling (Sekhwal et al., 2013). Molecular evidence suggests the involvement of several pentatricopeptide repeat proteins in both biotic and abiotic stress responses (Sharma & Pandey, 2016). Ribonuclease proteins are promising candidates for wheat defense, with distinct expression patterns observed in both susceptible and resistant cultivars (Khaleghi et al., 2021).

Another candidate gene encoding an ankyrin repeat‐containing protein is located in the plasma membrane and may function as a nonselective, Ca2+‐permeable cation channel that mediates resistance (Kolodziej et al., 2021). FORMIN proteins, which contain FORMIN Homology domain 1 and FORMIN Homology domain 2, play crucial roles in organismal growth and development (B. Li et al., 2023). EF‐hand domain‐containing proteins, which are involved in plant growth and development, are also involved in responses to abiotic stresses (e.g., salt, drought, cold, and abscisic acid) as well as biotic stresses (e.g., bacterial, viral, and fungal pathogens) (Kaur & Upadhyay, 2022).

MADS‐box transcription factors are a well‐documented group of genes that regulate growth and development in plants (Ali et al., 2019; Smaczniak et al., 2012). These genes control diverse biological functions, including cell development, signal transduction, responses to biotic and abiotic stresses, the development of vegetative organs, the regulation of flowering and anthesis, the development of meristems and flower organs, ovule and embryo development, dehiscence zone formation, and the ripening of fruits and seeds (Ali et al., 2019; Alvarez‐Buylla et al., 2000; Messenguy & Dubois, 2003; Moore et al., 2002; Parenicova et al., 2003; Saedler et al., 2001; Samach et al., 2000; Samaczniak et al., 2012). These promising candidate genes merit further investigation through cloning, characterization, and exploration of their potential for manipulation via overexpression, gene silencing, or gene‐editing approaches.

## 5 CONCLUSION AND FUTURE DIRECTIONS

SB continues to pose a significant challenge to wheat production across major growing regions, especially in South Asia, where recurrent epidemics result in substantial yield losses and compromised grain quality. This study confirmed the quantitative nature of SB resistance by identifying several significant SNPs distributed across diverse chromosomal regions. Seven stable MTAs, explaining 3.86%–18.17% of the phenotypic variation, were consistently detected under artificially inoculated field conditions, along with multiple resistant genotypes. These genotypes, notably BGD54, IND56, and BGD55, represent valuable sources of resistance that can be exploited in breeding programs. In silico functional annotation of the SNP‐associated genomic regions led to the identification of candidate genes involved in disease resistance pathways, including those encoding potassium transporters, zinc finger proteins, glutathione S‐transferases, and LRR proteins. Future research should focus on the functional validation of the identified candidate genes through transcriptomic, proteomic, and gene‐editing approaches to elucidate their precise roles in defense responses. Additionally, fine‐mapping and positional cloning of stable MTAs will increase the resolution of the associated loci and facilitate their deployment in MAS. Integrating these genomic tools into conventional breeding pipelines will accelerate the development of high‐yielding, SB‐resistant wheat cultivars tailored for vulnerable regions, thereby ensuring sustainable wheat production under biotic stress conditions.

## AUTHOR CONTRIBUTIONS

**Nikita Aggarwal**: Writing—original draft. **Xinyao He**: Data curation; investigation; validation; writing—review and editing. **Mukesh Rathore**: Data curation; methodology; writing—review and editing. **Farkhandah Jan**: Data curation; writing—review and editing. **Vikas Gupta**: Methodology; writing—review and editing. **Reyazul Rouf Mir**: Data curation; supervision; writing—review and editing. **Pawan K. Singh**: Conceptualization; funding acquisition; resources; writing—review and editing.

## CONFLICT OF INTEREST STATEMENT

The authors declare no conflicts of interest. The Technical Editor (TE) declares prior co‐authorship with both the Associate Editor (AE) and the Corresponding Author of this manuscript. The TE, AE, and Corresponding Author also serve on the Editorial Board of The Plant Genome. In accordance with the journal's editorial policies and COPE guidelines, appropriate measures were implemented to manage this potential conflict. The manuscript underwent independent peer review, and editorial decisions were made in line with standard journal procedures.

## Supporting information




**Figure S1**: Manhattan plots for spot blotch resistance generated using the GLM, MLM, MLMM, FarmCPU and CMLM model across environments (SB20 and SB21).
**Figure S2**: Manhattan plots for spot blotch resistance generated using the GLM, MLM, MLMM and CMLM model across environments (SB21 and SBAv).
**Figure S3**: Q–Q plots for spot blotch resistance generated using the GLM, MLM, MLMM, FarmCPU and CMLM model across environments (SB20, SB21 and SBAv).

**Table S1**: Mean days to heading, plant height and AUDPC for spot blotch disease across environments (years) in bread wheat genotypes.
**Table S2**: List of significant markers (p < 0.001) for spot blotch identified via GLM for each environment and the mean data.
**Table S3**: List of significant markers (p < 0.001) for spot blotch identified via CMLM for each environment and the mean data.
**Table S4**: List of significant markers (p < 0.001) for spot blotch identified via MLM for each environment and the mean data.
**Table S5**: List of significant markers (p < 0.001) for spot blotch identified via MLMM for each environment and the mean data.
**Table S6**: List of significant markers (p < 0.001) for spot blotch identified via FarmCPU for each environment and the mean data.
**Table S7**: List of significant markers (p < 0.001) for days to heading identified via GLM for each environment and the mean data.
**Table S8**: List of significant markers (p < 0.001) for days to heading identified via CMLM for each environment and the mean data.
**Table S9**: List of significant markers (p < 0.001) for days to heading identified via MLM for each environment and the mean data.
**Table S10**: List of significant markers (p < 0.001) for days to heading identified via MLMM for each environment and the mean data.
**Table S11**: List of significant markers (p < 0.001) for days to heading identified via FarmCPU for each environment and the mean data.
**Table S12**: List of significant markers (p < 0.001) for plant height identified via GLM for each environment and the mean data.
**Table S13**: List of significant markers (p < 0.001) for plant height identified via CMLM for each environment and the mean data.
**Table S14**: List of significant markers (p < 0.001) for plant height identified via MLM for each environment and the mean data.
**Table S15**: List of significant markers (p < 0.001) for plant height identified via MLMM for each environment and the mean data.
**Table S16**: List of significant markers (p < 0.001) for plant height identified via FarmCPU for each environment and the mean data.
**Table S17**: Information for stable marker‐trait associations associated with Plant height and Days to Heading identified via GWAS
**Table S18**: Details on putative candidate genes associated with stable and significant MTAs for studied traits
**Table S19**: Tsn1 allele status across the association panel, highlighting genotypes carrying the functional Tsn1 allele.

## Data Availability

All data generated or analyzed during this study are included in this published article and its supplementary information files. The genotypic data are available at https://hdl.handle.net/11529/10548634.
