# Supplementary material for: Identification of resistance sources and genomic regions regulating spot blotch resistance in Asian bread wheat (Triticum aestivum L.) via genome‐wide association study
Source: Plant Genome. 2026 Mar 27;19(2):e70228. doi: 10.1002/tpg2.70228 (PMC13022948; doi:10.1002/tpg2.70228)
Supplement: Supplementary file 1 — Figure S1: Manhattan plots for spot blotch resistance generated using the GLM, MLM, MLMM, FarmCPU and CMLM model across environments (SB20 and SB21). Figure S2: Manhattan plots for spot blotch resistance generated using the GLM, MLM, MLMM and CMLM model across environments (SB21 and SBAv). Figure S3: Q–Q plots for spot blotch resistance generated using the GLM, MLM, MLMM, FarmCPU and CMLM model across environments (SB20, SB21 and SBAv). [file TPG2-19-e70228-s002.docx]

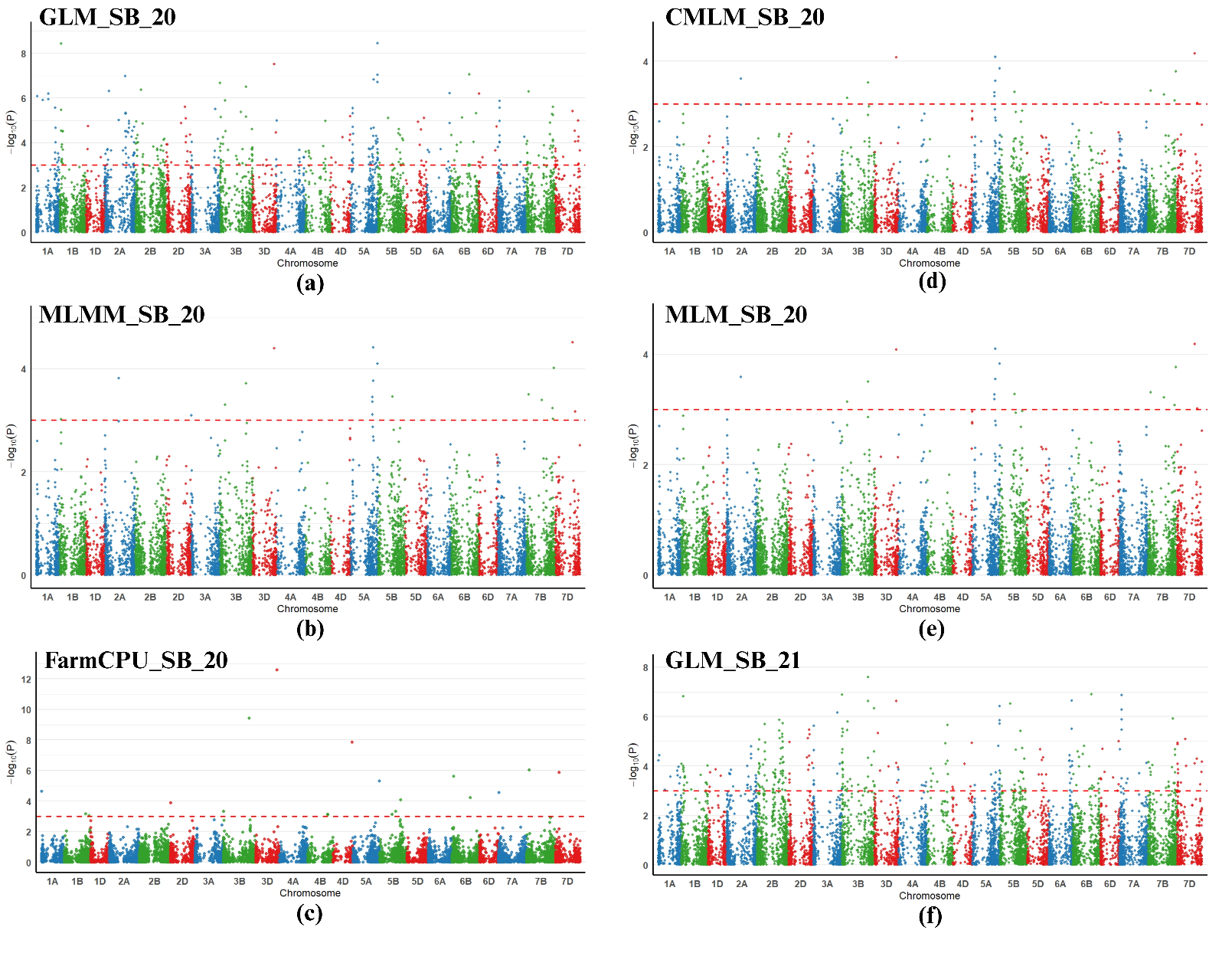
**Figure S1:** Manhattan plots for spot blotch resistance generated using the GLM, MLM, MLMM, FarmCPU and CMLM model across environments (SB20 and SB21).


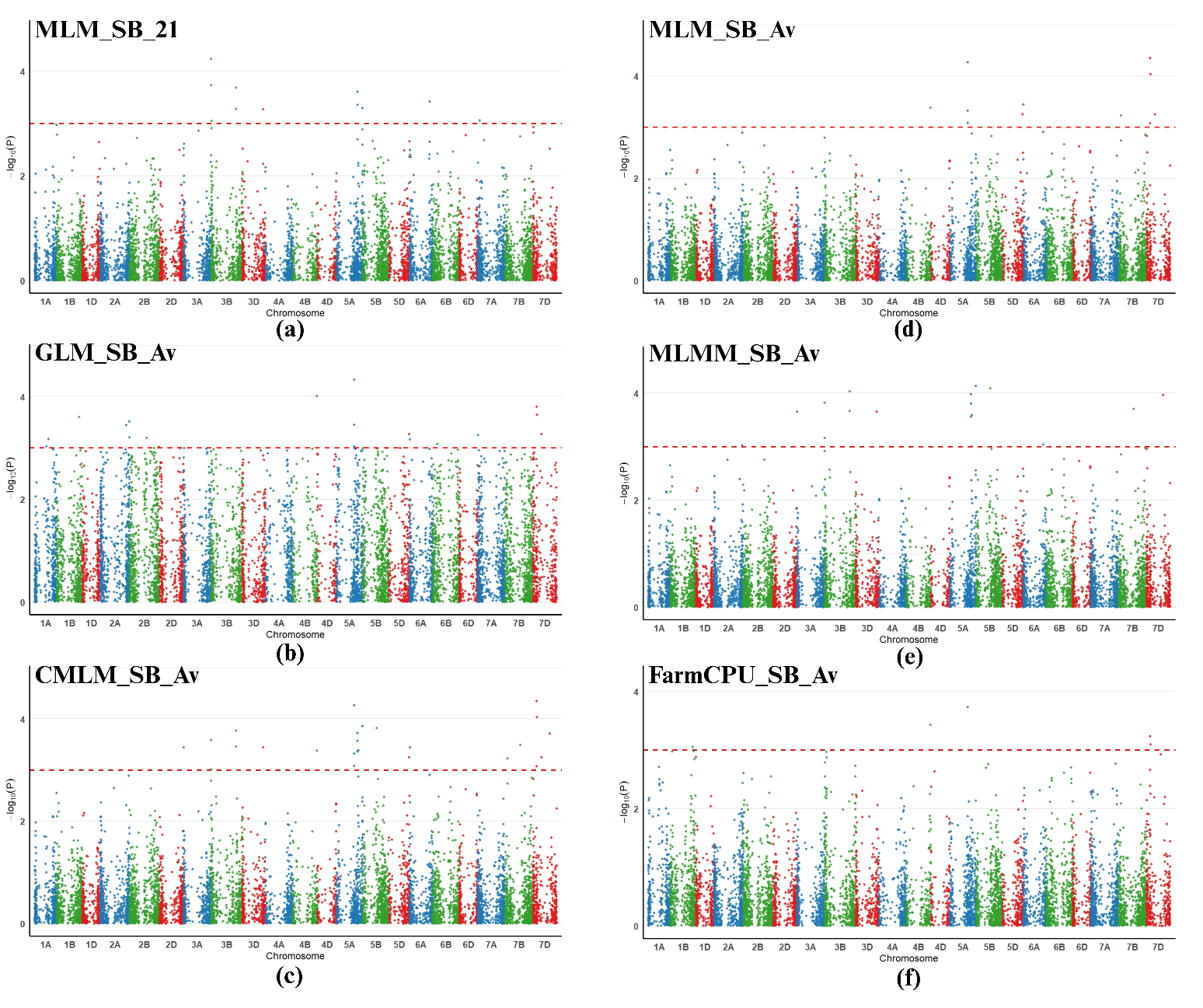
**Figure S2:** Manhattan plots for spot blotch resistance generated using the GLM, MLM, MLMM and CMLM model across environments (SB21 and SBAv).


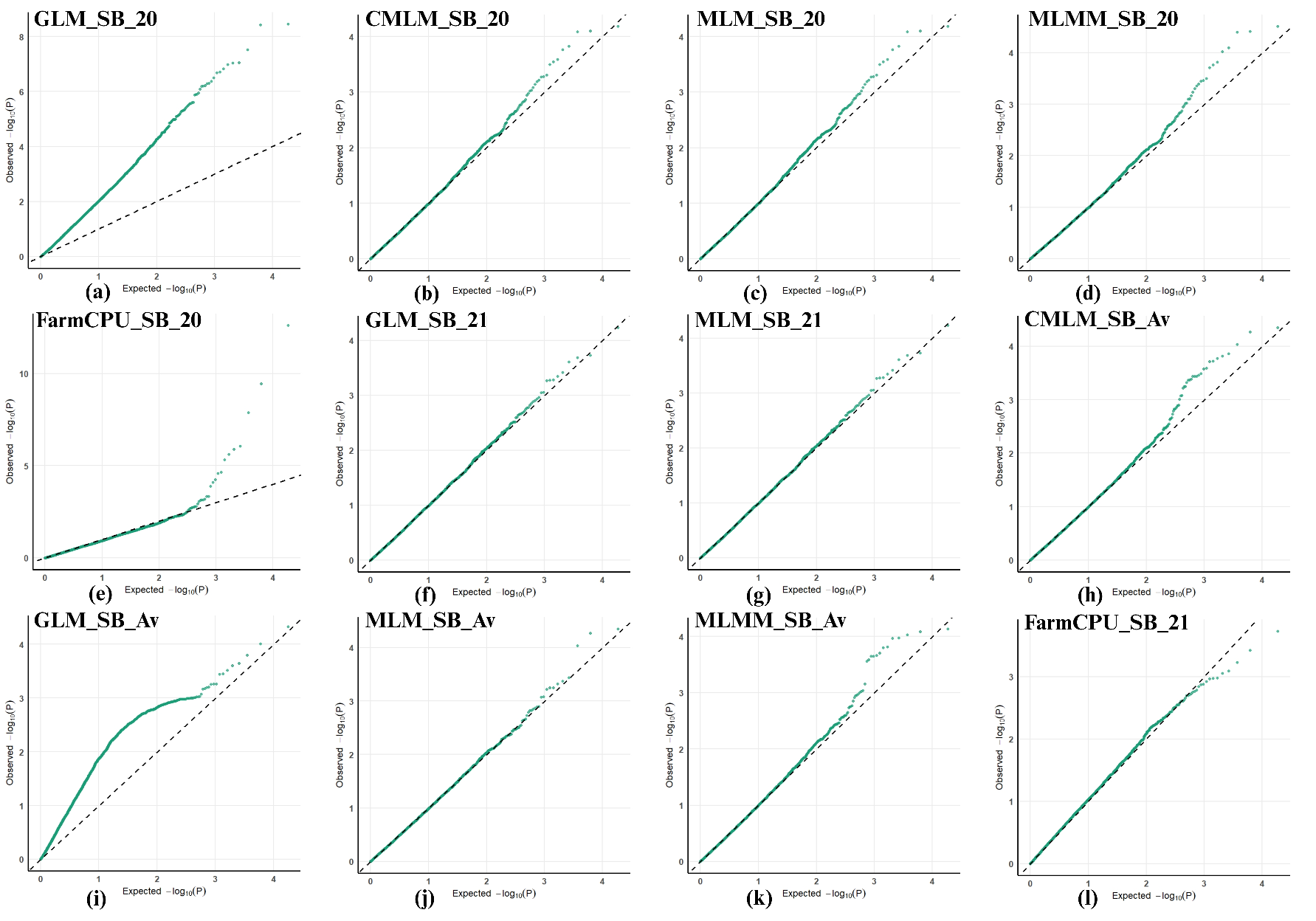
**Figure S3:** Q–Q plots for spot blotch resistance generated using the GLM, MLM, MLMM, FarmCPU and CMLM model across environments (SB20, SB21 and SBAv).
